# Enhanced Matching of Children’s Faces in “Super-Recognisers” But Not High-Contact Controls

**DOI:** 10.1177/2041669520944420

**Published:** 2020-07-26

**Authors:** Sarah Bate, Rachel Bennetts, Ebony Murray, Emma Portch

**Affiliations:** Department of Psychology, Bournemouth University, Poole, United Kingdom; Division of Psychology, College of Health and Life Sciences, Brunel University London; Department of Psychology, Bournemouth University, Poole, United Kingdom

**Keywords:** face recognition, individual differences, super-recognisers, face matching

## Abstract

Face matching is notoriously error-prone, and some work suggests additional difficulty when matching the faces of children. It is possible that individuals with natural proficiencies in adult face matching (“super-recognisers” [SRs]) will also excel at the matching of children’s faces, although other work implicates facilitations in typical perceivers who have high levels of contact with young children (e.g., nursery teachers). This study compared the performance of both of these groups on adult and child face matching to a group of low-contact controls. High- and low-contact control groups performed at a remarkably similar level in both tasks, whereas facilitations for adult and child face matching were observed in some (but not all) SRs. As a group, the SRs performed better in the adult compared with the child task, demonstrating an extended own-age bias compared with controls. These findings suggest that additional exposure to children’s faces does not assist the performance in a face matching task, and the mechanisms underpinning superior recognition of adult faces can also facilitate the child face recognition. Real-world security organisations should therefore seek individuals with general facilitations in face matching for both adult and child face matching tasks.

The matching of facial identity across two simultaneously presented images is a notoriously difficult task, even under the simplest of circumstances (Burton et al., 2010; Megreya & Burton, 2006). The performance declines for unfamiliar compared with familiar face pairs (Bruce et al., 2001), and when task demands are increased (e.g., viewpoint varies in the two images: Estudillo & Bindemann, 2014; or there are long time periods between the two image captures: White et al., 2014). Such tasks are fundamental in policing and security settings, where officers are frequently required to decide whether two instances of a face represent the same identity. In the last decade, there has been increasing interest in addressing this issue via real-world deployment of individuals with proficient face recognition skills (Bate & Dudfield, 2019)—so-called super-recognisers (SRs; Bennetts et al., 2017; Russell et al., 2009). However, identifying individuals who are likely to perform well on these tasks is complicated by the fact that matching performance can vary substantially between individuals (Bruce et al., 2018) and can be considerably impacted by the category of faces that are being matched.

It is well established that most individuals show poorer performance when asked to identify faces of a different ethnicity than their own, and that this tendency can be reduced with exposure to faces of that ethnicity (Meissner & Brigham, 2001). These findings support the suggestion that facilitated face matching performance depends on previous exposure to relevant faces—a consideration that may apply not only to ethnicity but also to the *age* of target faces. Indeed, in typical perceivers, evidence suggests that adult face memory performance is reduced for young children’s faces (Anastasi & Rhodes, 2005; Chance et al., 1986; Rhodes & Anastasi, 2012) but may be facilitated in individuals who frequently have contact with children (e.g., nursery teachers: de Heering & Rossion, 2008; Harrison & Hole, 2009; Rhodes & Anastasi, 2012).

It is less clear whether facial age also influences unfamiliar face matching tasks, as some findings suggest that age-related biases are highly sensitive to task demands (e.g., Proietti et al., 2015, 2019). However, although only two studies to date have investigated the performance of typical adults when matching children’s faces, difficulties were apparent in both. White et al. (2015) found that participants were poorer at matching instances of children’s, as opposed to adult’s, faces when target photographs were taken at disparate time points (over 6–13 years), and presented simultaneously in a one-in-eight array. In a simultaneous matching task, Kramer et al. (2018) also found poorer performance when pairs comprised two photographs of infants (Experiment 1) or one infant and one child face (Experiments 2 and 3), than when pairs comprised two adult faces.

While the findings of both White et al. (2015) and Kramer et al. (2018) concur with the wider face recognition literature that adults struggle with the recognition of children’s faces, it is unclear how this problem may be addressed in occupational settings, where accurate identification is imperative for child protection. Indeed, increasing reports of child trafficking (National Crime Agency, 2018) make accurate child-to-identity document matching a fundamental issue, and many international security teams are also responsible for the identification of children pictured in indecent online images. Previous work indicates that two groups of individuals may be the best candidates for such tasks: SRs who are known to perform extremely well at the matching of adult faces or typical perceivers who have high levels of contact with young children.

At first sight, identification of individuals who are particularly good at face recognition is an inviting solution to the high error rate in unfamiliar face matching tasks. However, very recent work has begun to identify limitations in the deployment of SRs (Bate, Portch, et al., 2019). Some result from the protocols used to identify these individuals: Psychometric standard tests of face recognition ability may tap different processes to those required by real-world tasks, some individuals may be only proficient at particular tasks, and one-off assessments can obscure considerable fluctuation in the performance (Bate et al., 2018, Bate, Frowd, et al. 2019). Recent evidence also suggests that the skills of SRs may be limited to particular facial stimuli. Bate, Bennetts, et al. (2019) found that the face matching performance of Caucasian SRs did not extend to the faces of other ethnicities and was no better than non-SR native perceivers.

Only one study to date has examined the performance of SRs when recognising children’s faces. Belanova et al. (2018) compared SR and control performance in a sequential matching task where pairs comprised two infant faces. Findings mirrored those of Bate, Bennetts, et al. (2019) for other-ethnicity faces: SRs not only outperformed typical perceivers in the task but also showed relative decrements in the performance for other-age faces. However, as the paradigm involved a memory component, it is unknown how SRs will fare in a simultaneous matching task. Furthermore, Belanova et al. did not include a high-contact control group, and the relative importance of exposure compared with general face recognition ability cannot be gauged. This issue is further complicated by the failure of all existing studies to control for potential confounds in task difficulty. Not only are there morphological differences in adult versus child faces that may account for task difficulty irrespective of exposure, the relative calibration of comparable tasks has not been addressed. That is, it is unclear whether any differences in the performance are merely artefacts resulting from biases at the phase of image selection and pairings, rather than genuine differences in the ability to recognise faces from different age groups.

The current investigation aimed to address this issue. In the first study to date, the performance of SRs was assessed on simultaneous face matching tasks that used adult versus child faces. To address the issue of exposure, all SRs reported low levels of contact with young children, and their performance was compared with typical perceivers who had experienced high or low levels of contact with children. To remove potential confounds in task difficulty, we used two matching tests that had previously been calibrated to an equal difficulty level in a large number of control participants. This design enabled us to directly address the question of whether SRs or typical perceivers with high exposure to children’s faces are the best candidates to match facial images of unfamiliar children. In addition, as previous work indicates inconsistencies in individual SRs’ performance both within and between tasks, we employed a case-by-case approach to examine whether the top matchers of adult faces are also the best candidates for the matching of children’s faces.

## Method

### Participants

Three groups of adult Caucasian females took part in this study, all aged between 18 and 50 years. We only recruited female participants because (a) many more women than men are employed in nursery and primary school settings, making it difficult to recruit balanced numbers of males and females; and (b) it could be claimed that females are more interested in, or perhaps attend to, the faces of young children to a greater extent than males. We therefore held gender constant across all three groups.

One group of participants contained 20 nursery and primary school teachers (*M*age = 33.9 years, standard deviation [*SD*] = 8.1) who had all worked without career breaks for at least 2 years immediately prior to participation in this study (these participants are subsequently referred to as “high-contact controls”). The second group also contained 20 participants (*M*age = 30.1 years, *SD* = 9.0) and recruited from our departmental participant pool. All participants in this group reported low levels of contact with young children at the time of recruitment (subsequently referred to as “low-contact controls”), returning low scores (*M* = 1.60, *SD* = 0.50) on a Likert-type scale ranging from 1 (*no contact with young children*) to 5 (*high levels of contact with young children*). This control sample size was calculated to give 80% power to detect moderate-to-large between-subject main effects (*d* > 0.72) and small-to-moderate within-subject main effects (*d* > 0.34) and interactions (*d* > 0.36) in the analysis of the control groups (power calculations carried out in G*Power 3.1). The effect size for within-subject main effects (i.e., the comparison between performance on child and adult matching tests) is comparable to the effects obtained in a meta-analysis of the own-age bias (*g* = 0.37; Rhodes & Anastasi, 2012) and substantially lower than effects reported for comparable matching tasks (e.g., *d* = 1.17; White et al., 2015 Experiment 1); the effect size for the interaction (i.e., Contact × Face Age) is substantially lower than effects reported in the previous literature (e.g., *d* = 0.70; Harrison & Hole, 2009).

Ten SRs (*M*age = 39.0 years, *SD* = 7.3) also participated (see Table 1). The sample size for the SR group was calculated as having 80% power to detect large main effects (*d* > 1.2), for both within-subjects and between-subjects comparisons. All SRs had obtained scores that surpassed those of control participants by at least 1.96 *SD*s (norms were taken from Bate et al., 2018) on two tests of face recognition: the extended form of the Cambridge Face Memory Test (CFMT+: Russell et al., 2009) and the Models Memory Test (Bate et al., 2018). While the CFMT+ is a dominant test of face memory that is typically used for SR screening (e.g., Bobak et al., 2016), the Models Memory Test is a new, more challenging test of face memory that adopts the CFMT+ paradigm (see Bate et al., 2018). All SRs reported low levels of contact with young children, returning scores of 1 or 2 on the Likert-type scale specified earlier. High- and low-contact control participants (but not SRs) received a small financial incentive to encourage motivation. Ethical approval for the investigation was granted by the institutional ethics committee.

**Table 1.** Control Mean Percentage Accuracy (*SD*) for the CFMT+ and MMT, With Demographic Information and Individual *z* Scores for Each SR.

	Controls	SR1	SR2	SR3	SR4	SR5	SR6	SR7	SR8	SR9	SR10
Gender	20×M, 20×F	F	F	F	F	F	F	F	F	F	F
Age	33.6 (10.1)	30	48	42	44	35	46	31	48	32	34
CFMT+	67.77 (9.92)	2.46	2.26	2.46	2.26	2.46	2.66	2.36	3.05	2.36	2.46
MMT	53.25 (14.06)	2.14	2.30	2.77	2.06	2.69	2.14	2.93	2.22	2.61	2.61

*Note.* Chance is 33.33% on the CFMT+ and 25% on the MMT. Control norms (*N* = 40) are taken from Bate et al. (2018): cutoffs are set at 1.96 *SD*s from the control mean. SR = super-recogniser; CFMT+ = Cambridge Face Memory Test; MMT = Models Memory Test.

### Materials

Two face matching tests were used: one contained Caucasian adult faces (aged 20–35 years) and the other used Caucasian children’s faces (aged 1–5 years). The adult face matching test was developed in our laboratory for previous work (the Pairs Matching Test, Bate et al., 2018) and is sufficiently calibrated to discriminate between top-end performers. This task assesses participants’ ability to match simultaneously presented pairs of faces over 48 trials: half match in identity and the remainder display two different individuals. As each “match” trial contains two different images of the same individual, a total of 96 different images (taken from 72 different identities) are used in the test, with 12 male and 12 female pairs in each condition. All images were downloaded from Google image searches and cropped to display the entire face from the neck upward. Mismatched faces were paired according to their perceived similarity to each other (as informally judged by the experimenter making the pairings, based on basic similarities between faces, e.g., hairstyle and perceived age; see Bate et al., 2018), and all images were adjusted to 10 cm in width and 14 cm in height. Trials were displayed in a random order until responses were made via key presses (the “S” key for “same” response, and the “N” key for “different” responses). While no time limit was imposed, participants were instructed to make their responses as quickly and accurately as possible.

The exact same parameters and protocols apply to the test using young children’s faces. This task was developed as part of a previous project that aimed to examine the consistency of performance across multiple versions of the Pairs Matching Test (Bate, Frowd, et al., 2019). For this reason, each version of the task was calibrated to be consistent in difficulty level via materials analyses performed on data collected from a large sample of typical perceivers. In effect, this calibration removed any inequalities in task difficulty that may result from any underlying own-age bias or biases in stimuli selection and pairing. Thus, if the SR or high-contact control group displayed any difference in the performance between the two tasks, this would indicate a difference in the own-age bias compared with typical perceivers.

### Procedure

All participants completed the two matching tests online. The low- and high-contact control groups completed both tests within the same session in a randomised counter-balanced design. As SRs had already completed the adult task during their initial screening session, we carried these scores over to this study to avoid exposure effects. They completed the children’s version approximately 1 year after initial screening occurred.

### Statistical Analyses

For each test, scores for all participants were calculated in terms of hits (the number of correct “same” responses) and correct rejections (the number of correct “different” responses) and summed for overall accuracy. These data were also used to calculate signal detection theory measures of recognition. Visual inspection of the hit and correct rejection data revealed departures from normality on several measures, and the Shapiro–Wilk statistic confirmed significant departures from normality for correct rejections on the adult test, and for hits on the child test, *W*(50) < .95, *p*s < .032. This was primarily driven by skewness, which varied between −0.31 and −0.57 (minimal-to-moderate negative skew). Due to these departures from normality, we used a nonparametric measure of sensitivity (*A*) and bias (*b*; Zhang & Mueller, 2005). The measure *A* ranges from 0 (*chance performance*) to 1 (*perfect performance*); the measure *b* is used as an indicator of response bias (i.e., whether the participant has a tendency to say that the target is present or absent; Macmillan & Creelman, 2005). A score of 0 indicates a neutral response criterion, whereas a positive score indicates conservative responding (a tendency to indicate that a target was not present) and a negative score indicates more liberal responding (a tendency to indicate that a target was present).

These measures were initially used to compare performance at the group-level between high- and low-contact controls. We carried out traditional and Bayesian analyses of variance (ANOVAs) to draw conclusions about the strength of evidence for differences (or lack of differences) between the control groups. For Bayesian analyses, a Cauchy prior distribution was used to estimate Bayes factors (BFs), centred on 0 and with scale parameters of *r* = .5 (Krypotos et al., 2017; Quintana & Williams, 2018). All ANOVA were carried out in JASP (JASP Team, 2020).

Because there was a much smaller sample of SRs, and we expected these individuals to display heterogeneous patterns of the performance, we analysed their performance at the single-case level. Crawford and Garthwaite’s (2002) modified *t* tests for single-case comparisons were used to determine whether each individual significantly outperformed controls on the two tests using the *singlism.exe* programme. Based on the size of the control group and the calculations presented by Crawford and Garthwaite (2006), we estimate that our statistical power to detect effects greater than 2 *SD*s from the mean was between 0.50 and 0.60.

## Results

### Control Participants

A 2 (Group [high-contact, low-contact]) × 2 (Version [adult, child]) mixed-factorial ANOVA on *A* did not result in a significant interaction, *F*(1,38) = 0.110, *p* = .742, ηρ^2^ = .01 (see Table 2). Neither the version nor group main effects were significant, *F*(1,38) = 0.017, *p* = .896, ηρ^2^ = .01, and *F*(1,38) = 0.463, *p* = .501, ηρ^2^ = .01, respectively. The same ANOVA was also run on *b* (bias): neither the interaction nor the version or group main effects were significant, *F*(1,38) = 0.001, *p* = .983, ηρ^2^ = .01, *F*(1,38) = 0.355, *p* = .555, ηρ^2^ = .01, and *F*(1,38) = 0.198, *p* = .659, ηρ^2^ = .01, respectively (see Table 2).

**Table 2.** Mean (*SD*) Performance on the Two Matching Tests for High- and Low-Contact Control Participants.

	High-contact controls		Low-contact controls	
	Adult faces	Children’s faces	Adult faces	Children’s faces
*A*	.74 (.08)	.75 (.08)	.76 (.10)	.76 (.08)
Hits (%)	58.66 (15.33)	63.13 (15.37)	66.04 (17.17)	62.08 (17.25)
CRs (%)	68.33 (15.44)	72.29 (12.04)	71.88 (17.41)	73.75 (13.59)
Overall (%)	67.08 (6.71)	67.71 (7.23)	68.96 (8.86)	67.92 (8.61)
*b* (bias)	1.16 (0.55)	1.21 (0.40)	1.23 (0.63)	1.28 (0.56)

*Note.* CR = correct rejection.

The Bayesian ANOVA on *A* revealed moderate evidence that the null model was favoured more than the model including version (BF_01_ = 4.28), but little evidence that it was favoured more than the model including group (BF_01_ = 2.87; this may indicate that the data were not sensitive enough to reliably detect group differences in sensitivity). However, the main point of interest was the interaction between version and group. There was very strong evidence that the null model was favoured more than the model including main effects and interactions (BF_01_ = 38.65); furthermore, the main effects model alone (BF_01_ = 12.36) was preferred to the model including interaction by a factor of 3.13. The Bayesian ANOVA for *b* showed a similar pattern of findings: little evidence for the null model being favoured over the model including group (BF_01_ = 2.36), moderate evidence for the null model being preferred over version (BF_01_ = 3.75), but strong support for the null when compared with models including the main effects and Group × Version interaction (BF_01_ = 27.99); the model including main effects alone was again preferred over the model including interactions, by a factor of 3.11. In short, the Bayesian ANOVA offers support for the null hypothesis (no interaction between contact and the performance on the adult and child tasks), which was the key effect of interest.

To independently examine the performance on matched and mismatched trials, a 2 (Group [high-contact, low-contact]) × 2 (Version [ adult, child]) × 2 (Response [hits, correct rejections]) mixed-factorial ANOVA was performed. There was no influence of participant group (see Table 2): the three-way interaction was nonsignificant, *F*(1, 38) = 0.017, *p* = .898, ηρ^2^ = .01, as were the two-way interactions with version, *F*(1, 38) = 0.282, *p* = .599, ηρ^2^ = .01, and response, *F*(1, 38) = 0.139, *p* = .712, ηρ^2^ = .01, and the group main effect, *F*(1, 38) = 0.286, *p* = .596, ηρ^2^ = .01. Version and response did not interact, *F*(1, 38) = 3.735, *p* = .061, ηρ^2^ = .09, and no significant main effect was found for either measure, *F*(1, 38) = 0.018, *p* = .895, ηρ^2^ = .01 and *F*(1,38) = 3.471, *p* = .070, ηρ^2^ = .08, respectively. Once again, the Bayesian ANOVA offered moderate support for the null model over the model including group alone (BF_01_ = 4.63) and strong support for the null hypothesis over all models containing a group-based interaction (BF_01_ = 8.43–131.03). Taken together with the *A* and *b* results, this provides strong evidence for the claim that there is no difference in the pattern of matching performance for high- and low-contact individuals across the two different versions of the test.

## Super-Recognisers

Given the remarkable similarity of high- and low-contact controls on the two matching tests, data were collapsed across all control participants for purposes of comparison to the SRs. This produced overall *A* control means of 0.75 (*SD* = 0.09) and 0.75 (*SD* = 0.08) for the adult and children’s tasks, respectively. There was no correlation between the performance on the two tasks for the collapsed control group (*N* = 40, *r* = .195, *p* = .228).

**Figure 1.**
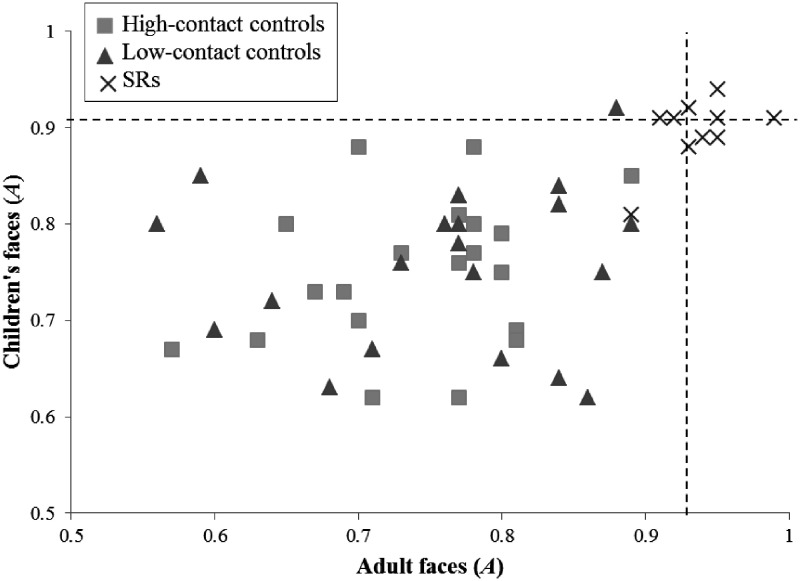
Performance (A) of High- and Low-Contact Controls and SRs on the Matching of Adult and Child Faces. Dotted lines represent 1.96 *SD*s from the overall control mean. SR = super-recogniser.

The performance of 9 of the 10 SRs was consistently high on both tests, with *A z* scores ranging from 1.78 to 2.67 for adult faces and from 1.63 to 2.38 for children’s faces. One individual performed less well on both tests, achieving *z* scores of 1.56 and 0.75, respectively (see Figure 1). Modified *t* tests confirmed that five SRs outperformed controls on the adult test. Only two SRs (SR07 and SR10) outperformed controls on the child test (see Table 3), although only SR10 also significantly outperformed controls on the adult test.

**Table 3.** Modified *t* Tests for Single-Case Comparisons (Crawford & Garthwaite, 2002) for the Performance of 10 Individual SRs in Relation to the 40 Control Participants, on the Adult (Control *M* = 0.75, *SD* = 0.09) and Child (Control Mean = 0.75, *SD* = 0.08) Face Matching Tests.

	*A*	*t*	*p*	*z*-cc	Percentage populationless extreme
Adult test					
SR1	.93	1.98	.06	2.00	97.23
SR2	.94	2.09	.04	2.11	97.82
SR3	.95	2.20	.03	2.22	98.29
SR4	.91	1.76	.09	1.78	95.65
SR5	.92	1.87	.07	1.89	96.52
SR6	.99	2.63	.01	2.67	99.40
SR7	.93	1.98	.06	2.00	97.23
SR8	.95	2.20	.03	2.22	98.29
SR9	.89	1.54	.13	1.56	93.38
SR10	.95	2.20	.03	2.22	98.29
Child test					
SR1	.88	1.61	.12	1.63	94.17
SR2	.89	1.73	.09	1.75	95.41
SR3	.89	1.73	.09	1.75	95.41
SR4	.91	1.98	.06	2.00	97.23
SR5	.91	1.98	.06	2.00	97.23
SR6	.91	1.98	.06	2.00	97.23
SR7	.92	2.10	.04	2.13	97.88
SR8	.91	1.98	.06	2.00	97.23
SR9	.81	0.74	.46	0.75	76.84
SR10	.94	2.35	.02	2.38	98.79

*Note.* Raw scores for *A* and effect sizes (*z*-cc) are reported for each calculation, alongside the percentage of the population achieving less extreme scores than each individual SR.

Inspection of each individual’s performance indicates that every SR achieved a score that was numerically lower on the children’s test compared with the adult’s test; a paired-samples *t* test found that the performance was higher on the adult (*M* = 0.94, *SD* = 0.03) compared with children’s (*M* = 0.90, *SD* = 0.03) test for the SRs as a group, *t*(9) = 4.511, *p* = .001, *d* = 1.33. This effect held even when the low-performing SR was eliminated from the analysis, *t*(8) = 4.042, *p* = .004, *d* = 1.50. The size of this effect was then compared with the relative performance of controls, by subtracting *A* scores on the children’s test from *A* scores on the adult test. An independent samples *t* test confirmed that this difference was greater in SR (*M* = 0.04, *SD* = 0.03) compared with control (*M* = 0.00, *SD* = 0.11) participants, *t*(47.55) = 2.259, *p* = .029, *d* = 0.50. As found for controls, there was no difference between the two tests for response bias (*b*) in the SR group, *t*(9) = 0.790, *p* = .450, *d* = 0.26.

## General Discussion

This investigation examined the performance of SRs when matching children’s compared with adult’s faces, in relation to high- and low-exposure control groups. There was no indication of facilitated matching of children’s faces in the high-exposure group. While most SRs displayed very good matching performance for both adult and children’s faces, only five significantly outperformed controls on the adult task and only two on the children’s task. Critically, as a group, SRs’ performance was lower for child compared with adult faces, and this dissociated from control performance.

Our finding that scores were remarkably similar for high- and low-contact controls across adult and child faces is important. Supported by Bayesian analyses, this null finding indicates that increased contact with children is of no benefit in the simultaneous matching of children’s faces. Instead, because the two versions of the task were matched in difficulty, any underlying own-age bias in matching performance was equivalent for the high- and low-contact groups. It is possible that the own-age bias in face recognition is highly sensitive to task demands, and that increased exposure to particular types of face only has limited benefits. For instance, increased visual experience with children’s faces may assist with speeded judgments, where there is a benefit in looking at diagnostic sources of information earlier in processing, or when recalling a face from memory. Although participants were asked to respond as quickly as possible in this study, they nevertheless had unlimited time to match two simultaneously presented faces, and this may have allowed low-contact controls to equate the performance of their high-contact counterparts. Importantly, this finding demonstrates that experience with children’s faces may be of no benefit to real-world face matching scenarios where split-second judgments are typically not required, and instead, organisations should seek to use individuals with natural proficiencies in face matching performance.

Indeed, all but one of the SRs obtained consistently high scores when matching adult and child faces, performing within the top 7% of the population on both tasks, irrespective of whether single-case comparisons reached significance. It is possible a ceiling effect emerging from control norms made some SRs just miss the cutoff for superior performance, leaving little room for even a small number of errors. It is therefore possible that more of the SR cohort would reach the superior range in a more difficult task. Nevertheless, the finding of generally high scores on both tasks indicates that SRs with more general facilitations in unfamiliar face memory (i.e., the entry criterion for this study) are also very good candidates for matching tasks that involve children’s faces.

However, one caveat can be found in the single SR who did not perform well at either the adult or child matching task. As in most other SR studies, the inclusion criteria for this investigation were the performance on tests of unfamiliar face memory. Consistent with our previous work (Bate et al., 2018; Bate, Frowd, et al., 2019), the facilitated performance of SR9 was restricted to face memory, and this individual achieved the lowest scores in the two matching tasks. Given unfamiliar face memory and matching represent quite different tasks, this finding reinforces existing suggestions that real-world SR screening programmes should imitate the task in hand (Bate et al., 2018; Bate, Frowd, et al., 2019).

Yet, despite evidence of consistently high performance on both tasks, the SRs as a group achieved lower scores on the child compared with adult task, and this difference dissociated from control performance. Because the equal calibration in task difficulty accounted for an own-age bias in controls, this decline in SR performance indicates a more substantial own-age bias than indicated by the mean scores. This finding suggests that the perceptual mechanisms underpinning super recognition are more attuned to adult than child faces. This may be due to differences in processing style. It is possible that adults have a tendency to process child faces in a less holistic manner than adult faces (e.g., de Heering & Rossion, 2008), and SRs may rely on increased levels of holistic processing to achieve high levels of performance in adult face recognition tasks (e.g., Bobak et al., 2016; Russell et al., 2009).

 Nevertheless, most SRs still performed very well on the child task. Explanations of the own-age bias have sometimes invoked the idea of a face space (Valentine, 1991) that codes for dimensions of variability which are encountered frequently (e.g., faces of a similar age to oneself) and is less sensitive for dimensions that are no longer regularly encountered (e.g., children’s faces, once one reaches adulthood: Macchi Cassia et al., 2009). The current findings may indicate that SRs develop their face-space in a similar, but more efficient, way to typical individuals, leading to enhanced performance compared with controls even when viewing relatively unfamiliar classes of faces, but similar patterns of bias and limitation as those with typical face recognition abilities. To date, no work has examined the face-space of SRs, but based on our current findings and those of Bate, Bennetts, et al. (2019), we would expect any differences between SRs and typical perceivers to be quantitative (e.g., enhanced or more rapid effects of exposure in SRs), rather than qualitative (e.g., use of different dimensions between SRs and typical controls) in nature.

In sum, this study presents evidence of high matching performance for children’s faces in SRs who also excel at adult face matching. Despite evidence of an own-age bias in these individuals, performance was nevertheless at a high level, indicating that the processes which underpin super-recognition for adult faces are also likely deployed when matching child faces. In light of the finding that a high-exposure control group did not excel at the task, it is recommended that individuals with generally high face matching abilities are also deployed for real-world matching tasks that involve children’s faces.
